# Preparation and Properties of Lightweight Geopolymer by Bio-Based Foaming Agent

**DOI:** 10.3390/ma17133167

**Published:** 2024-06-27

**Authors:** Tianlei Wang, Yao Chen, Xiudang Jing, Xueping Wang, Lei Zhang, Peisen Yang

**Affiliations:** 1 School of Materials Science and Engineering, Tianjin Chengjian University, Tianjin 300384, China; wangtl@tcu.edu.cn (T.W.); 15822303588@163.com (Y.C.); 15732759005@163.com (X.J.); zhanglei@tcu.edu.cn (L.Z.); 2 Tianjin Zhaoyang Environmental Technology Group Co., Ltd., Tianjin 300384, China; 15822236758@163.com

**Keywords:** yeast, bio-based foaming agent, geopolymer, lightweight

## Abstract

Lightweight geopolymers have the advantages of a wide source of raw materials, chemical corrosion resistance, high mechanical strength and excellent durability, and are expected to replace traditional building insulation materials. In this paper, a green bio-based foaming agent with a small 1 h settlement distance, high average foaming multiple and low bleeding ratio was obtained by a Cetyltrimethylammonium Bromide/yeast solution. When the amount of Cetyltrimethylammonium Bromide is 0.50 wt%, the foam prepared by the yeast and Cetyltrimethylammonium Bromide solution exhibits the improved 1 h settlement distance, the large average foaming multiple, the small bleeding ratio and uniform foam size. Subsequently, a lightweight geopolymer based on metakaolin and fly ash (or silica fume) was successfully prepared by the bio-based foaming agent, and the effects of different foam content on the properties of the geopolymer, such as dry density, water absorption, thermal conductivity, compressive strength and morphology, were studied. With an increase in foam content, the dry density, thermal conductivity and compressive strength of the geopolymer gradually decrease, the water absorption increases, regardless of whether silica fume or fly ash are added. Herein, it is confirmed that the foaming agent based on yeast can be effectively used to prepare lightweight geopolymers, which can provide vast opportunities to turn into candidates for novel inorganic thermal insulation materials.

## 1. Introduction

Concrete has become the preferred material for construction engineering because of its versatility, adaptability and low cost [1,2]. The production process of ordinary Portland cement, one of the important components of concrete, requires limestone, clay, coal, fuels and other raw materials, which not only consumes a lot of natural resources and energy, but also emits harmful gases (such as CO_2_ and SO_2_), resulting in ecological destruction and environmental pollution [3,4,5,6]. Geopolymers, normally prepared by metakaolin and waste products (such as silicon fume, fly ash and tailings slag), have been considered a kind of green cementitious material with great potential, because their CO_2_ emission in the whole production process is only around one-fifth of that of ordinary Portland cement [7,8,9]. Therefore, geopolymers have gradually become the focus of researchers in recent years, as they are considered a sustainable and green material.

As a new type of inorganic thermal insulation material, lightweight geopolymers exhibit excellent mechanical and durability properties [10,11]. Jaya NA et al. [12] prepared a metakaolin-based geopolymer by chemical foaming with H_2_O_2_, and the compressive strength could reach 33.0 MPa after 28 days. El-Naggar et al. [13] used the powder residue from clay brick waste, slaked lime waste generated in the production of acetylene and waste aluminum trimmings from workshops to prepare a geopolymer. Due to the reaction of aluminum with alkalis, there were a number of pores formed inside the geopolymer matrix, and lightweight bricks with a bulk density in the 1000 kg/m^3^ were prepared; the samples containing 10% aluminum exhibited lower thermal conductivities, reaching values as low as 0.24 W/(m·K). Varying H_2_O_2_ and Tween 80 contents, a lightweight geopolymer with a bulk density of 471–1212 kg/m^3^, porosity of 36–86% and thermal conductivity of 0.11–0.30 W/(m·K) was obtained [14]. However, it has been found that the foam produced by chemical foaming agents is uneven and of different sizes, and some foaming agents produce harmful gases. Therefore, it is considered that the physical foaming agents, such as rosin resin foaming agents, synthetic surfactant foaming agents, protein foaming agents, etc., should be applied into the preparation of lightweight geopolymers [15,16]. Ibrahim et al. [17] successfully used polyoxyethylene alkyether sulfate as foaming agent to prepare a lightweight geopolymer. Although the physical foaming agents can improve on the shortcomings of the chemical foaming agent to a certain extent, a low foaming ratio and a high cost seriously limit their further large-scale production and application. Therefore, developing a new type of foaming agent with a high efficiency and environmental friendliness is very essential and significative.

Recently, the research on the preparation of lightweight materials by yeast foaming has aroused widespread attention [18,19,20]. A biological foaming technique through the reaction of yeast with starch in an aqueous ceramic suspension was applied to fabricate a red-clay-based porous ceramic. When the 11 wt% corn starch treated with 31.5% yeast was added in the ceramic slurry, the fired total porosity of the obtained porous ceramic could reach 70.3% [20]. Uhlířová T et al. [21] used yeast biological foaming to prepare highly porous alumina ceramics. It was found that the ceramics prepared usually have total porosity in the range of 78–84% and the porosity related to large pores is usually between 58% and 75%. A stable bio-based foaming agent can be obtained through yeast fermentation and biochemical reactions, without the need for external chemical reactions, while improving environmental pollution and human health hazards.

In this paper, a bio-based foaming agent was obtained by the yeast solution combined with Cetyltrimethylammonium Bromide (CTAB), and a lightweight geopolymer based on metakaolin and fly ash (or silica fume) was prepared by this bio-based foaming agent. As a result, this work will not only implement the development of environment-friendly and high-performance lightweight materials, but also realize the resource utilization of waste, so as to meet the requirements of low-carbon and sustainable development.

## 2. Experiments

### 2.1. Materials

Metakaolin was purchased from Chaopai Building Materials Technology Co., Ltd. (Inner Mongolia, China), with a fineness of 1250 mesh and an activity index greater than 110. The yeast came from the General Microbiology Center of China Microbial Species Preservation Administration Committee (Beijing, China). Silica fume was purchased from Changzhou Lingteng Composite Material Co., Ltd. (Changzhou, China), with a content of over 75% and a loss on ignition of 1.48%. The fly ash is Class F Grade II fly ash provided by a thermal power plant in Tianjin, with a loss on ignition of 3.1% and an activity index of 75%. Sodium silicate was purchased from Shandong Yusuo Chemical Technology Co., Ltd. (Heze, China). Peptone was purchased from Beijing Aobo Star Biotechnology Co., Ltd. (Beijing, China). Cetyltrimethylammonium Bromide (CTAB) and anhydrous glucose were purchased from Tianjin Damao Chemical Reagent Factory (Tianjin, China).

### 2.2. Preparation of Lightweight Geopolymer by Bio-Based Foaming Agent

The medium solution was composed of 1.0 g peptone, 0.5 g yeast immersion powder and 1.0 g anhydrous glucose in 100 mL water. The yeast was inoculated into the sterile medium solution at a ratio of 10%, and then cultured in a constant temperature oscillation incubator for 24 h. Then, the foam was prepared by the cultured yeast solution containing CTAB via the physical foaming method. Metakaolin and silica fume (or fly ash) were selected as the main raw materials to form the geopolymer using an alkaline activator with a modulus of 1.2. The alkali activator used an aqueous glass solution (modulus 3.3) and solid NaOH. As shown in Table 1, silica fume (or fly ash) and metakaolin were mixed in a container, and then the alkali activator with modulus of 1.2, polycarboxylate superplasticizer and water were poured into it. After mixing, foam was quickly added into the container to fully mix the bubbles with the slurry, and then the slurry was poured into the mold (50 × 30 × 30 mm). After curing for 48 h, the block was demoulded and continued to solidify at room temperature until the specified age.

A series of physical and mechanical tests were conducted, including compressive strength, dry density, water absorption and thermal conductivity, to determine the performance of the lightweight geopolymer. The type of the compressive strength testing machine (YAW-2000, Tenson, Jinan, China) was with a loading rate of 0.5 MPa/s, and the average value of three samples was taken for each test result. Three samples were randomly taken from each group and placed in a (60 ± 5) °C drying oven to dry until constant weight, so as to calculate the dry density of the samples. The samples with constant dried weight were put into a constant-temperature water tank with a temperature of 25 °C, and the added water was more than 30 mm higher than the samples. After soaking for 72 h, the water on the surface of the sample was removed and weighed to calculate its water absorption rate. The thermal conductivity was tested by the thermal conductivity tester (TC3000E, Xi’an Xiaxi Electronic Technology Co., Ltd., Xi’an, China), and the data were measured three times for each group of samples, and the average value was taken. A field emission scanning electron microscope (SEM, JSM-7800F, JEOL, Tokyo, Japan) and a super depth 3D microscope system (VHX-600E, KEYENCE, Osaka, Japan) were used to observe the morphological characteristics of the powder and foam, respectively.

## 3. Results and Discussion

**Figure 1:**
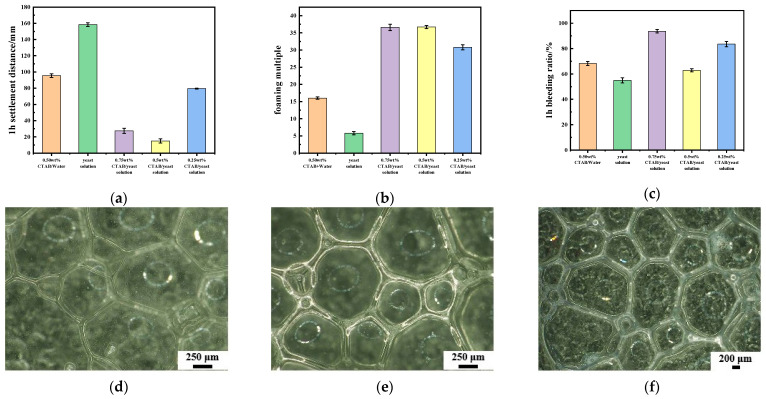
The 1 h settlement distance (**a**), average foaming multiple (**b**) and bleeding ratio (**c**) of the foaming agent; the morphology of the bio-based foaming agent when the CTAB content is 0.25 wt% (**d**), 0.50 wt% (**e**) and 0.75 wt% (**f**), respectively.

As shown in Figure 1a, the 1 h settlement distances of the foam prepared by yeast and CTAB are significantly improved compared with foam prepared by using 0.50 wt% CTAB alone with a water or yeast solution. This is mainly due to the increase in surface active molecules in the solution system, which reduces the surface tension of the solution and increases the stability of the foam. However, when the CTAB content is relatively low in the CTAB/yeast solution, the number of surface-active molecules and entrained solutions attached to the liquid film is small and the self-healing ability of the liquid film is poor, resulting in foam that is easily destroyed. When the content of CTAB is high, the large number of surface-active molecules attached to the liquid film make the foam gravity larger, leading to foam rupture. At the same time, the volume expansion of the CTAB/yeast solution is significantly improved. When the amount of CTAB is 0.50 wt%, the average foaming multiple of the foam prepared by the yeast and CTAB composite system is the largest (Figure 1b). In addition, Figure 1c shows the bleeding ratio of different foams, which is an important index to measure the stability of foam. When the content of CTAB is 0.25 wt%, the pore size of the foam is not uniform and the wall thickness is thin (~29 μm), resulting in a bleeding ratio of 83.6%, which makes it easy to defoam (Figure 1d). When the content of CTAB is 0.50 wt%, the foam has a uniform size, a wall thickness of around 38.24 μm and a suitable liquid volume between bubbles which makes the stability better (Figure 1e). As shown in Figure 1f, when the content of CTAB is 0.75 wt%, the pore size of the foam increases, the wall thickness of the foam is as high as 55.42 μm and the liquid between the bubbles accumulates, which causes the foam to burst easilu, thereby increasing its bleeding ratio. As a result, the content of 0.5 wt% CTAB with yeast solution was selected to prepare the bio-based geopolymer in subsequent experiments.

**Figure 2:**
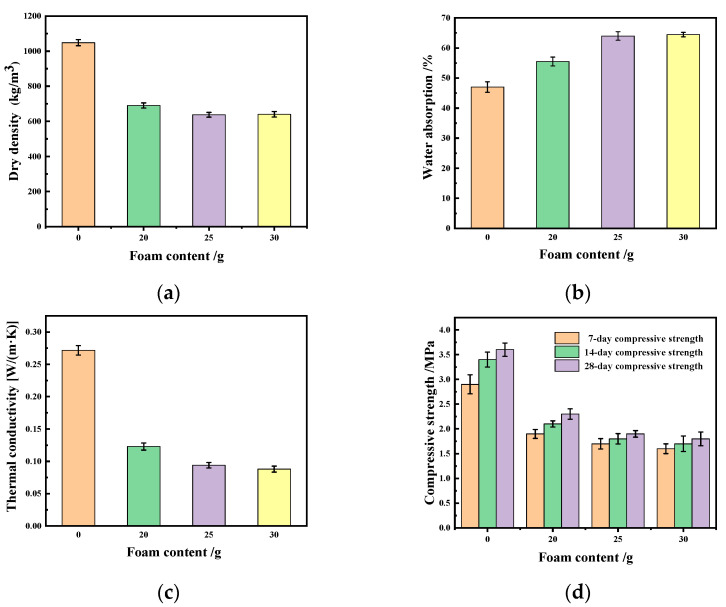
Dry density (**a**), water absorption (**b**), thermal conductivity (**c**) and compressive strength (**d**) of silica fume-based geopolymer.

**Figure 3:**
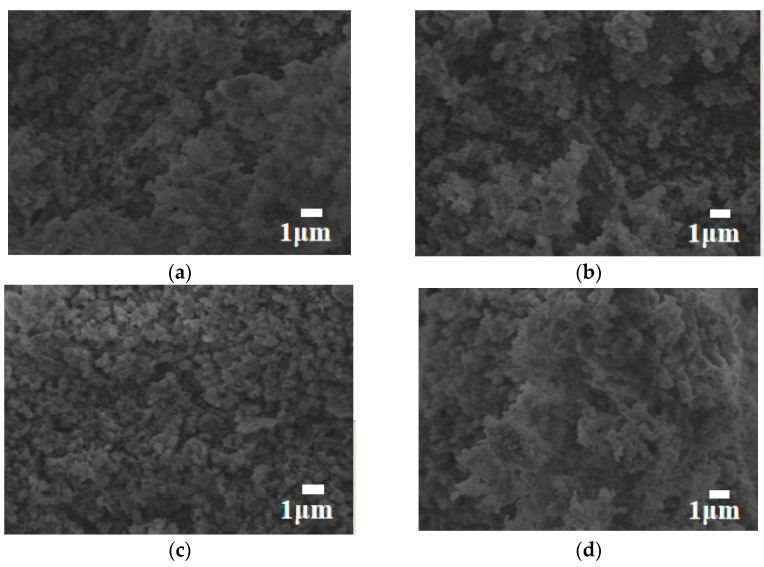
The morphology of the silica fume-based geopolymer when the foam content is 0 g (**a**), 20 g (**b**), 25 g (**c**) and 30 g (**d**), respectively.

As shown in Figure 2a, the dry density of a silica fume-based geopolymer without adding foam is 1050.7 kg/m^3^; with the increase in foam content, the dry density gradually decreases. But when the foam content increases to 30 g, the decrease in dry density is not obvious, because the geopolymer slurry has a high viscosity and large surface tension, which makes it difficult for foam to exist in the stable state, thus resulting in the defoaming phenomenon. As the dry density decreases, the porosity increases, resulting in an increase in the ability of water absorption (Figure 2b). When the foam content is 30 g, the water absorption of the lightweight silica fume-based geopolymer is as high as 64.6%. It is shown in Figure 2c that the thermal conductivity is positively correlated with the dry density of geopolymer. Compared to the high thermal conductivity of the silica fume-based geopolymer without adding foam [0.275 W/(m·K)], the thermal conductivity of the silica fume-based geopolymer obtained with the foam content of 20 g, 25 g and 30 g was 0.123 W/(m·K), 0.094 W/(m·K) and 0.088 W/(m·K), respectively. Meanwhile, it can be seen from Figure 2d that with the continuous hydration process, the compressive strength increases with the increase in age, whether foam is added or not. The compressive strength of the block gradually decreases with the increase in the foam content at the same curing age. In addition, the change in foam content has almost no effect on the microstructure of the silica fume geopolymer (Figure 3a–d). Compared with the silica fume geopolymer without foam, the N-A-S-H gel in the silica fume-based polymer with foam is more dispersed. This is mainly because the addition of foam can play a barrier role, making N-A-S-H gel tend to disperse. The presence of a large amount of dispersed cementitious products leads to an increase in pores in the sample, resulting in a decrease in compressive strength, an increase in water absorption and a beneficial impact on thermal insulation performance [17,22,23].

**Figure 4:**
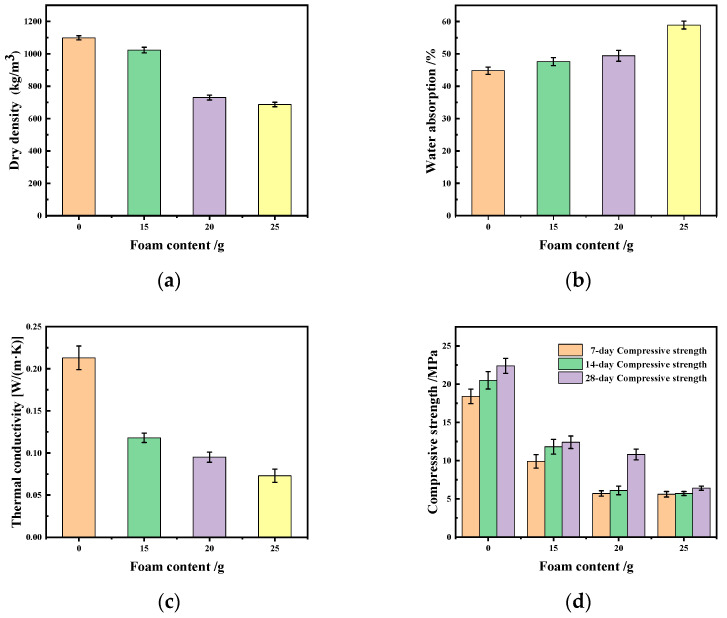
Dry density (**a**), water absorption (**b**), thermal conductivity (**c**) and compressive strength (**d**) of fly ash-based geopolymer.

In order to fully prove the universality of the bio-based foaming agent for the preparation of lightweight geopolymers, fly ash was used instead of silica fume in the experiment. The dry density reached 1107.3 kg/m^3^ without adding foam, and the dry density decreased gradually to 1023.8 kg/m^3^, 730.0 kg/m^3^ and 678.4 kg/m^3^, respectively, when the content of foam was 15 g, 20 g and 25 g (Figure 4a). The water absorption of the fly ash-based geopolymer increases, and the thermal conductivity decreases with the increase in foam content (Figure 4b,c). As shown in Figure 4d, the compressive strength of the samples gradually decreases with the increase in foam content, and increases with the growth in curing age. The dry densities of a lightweight geopolymer prepared using foaming agents such as a synthetic surfactant and plant surfactant are around 630 kg/m^3^, and the compressive strength is between 1.86 MPa and 2.66 Mpa. A lightweight geopolymer prepared with foaming agents such as nanometer aluminum oxide, synthetic surface, plant surface, aluminum powder, etc., has a dry density of around 600 kg/m^3^ and a compressive strength of 1.83 MPa–2.75 Mpa [24,25,26]. Our work uses a bio-based foaming agent, mixed with yeast solution and CTAB, to reduce the dry density of a fly ash-based lightweight geopolymer to 678.4 kg/m^3^, but the compressive strength remains around 6.4 Mpa.

**Figure 5:**
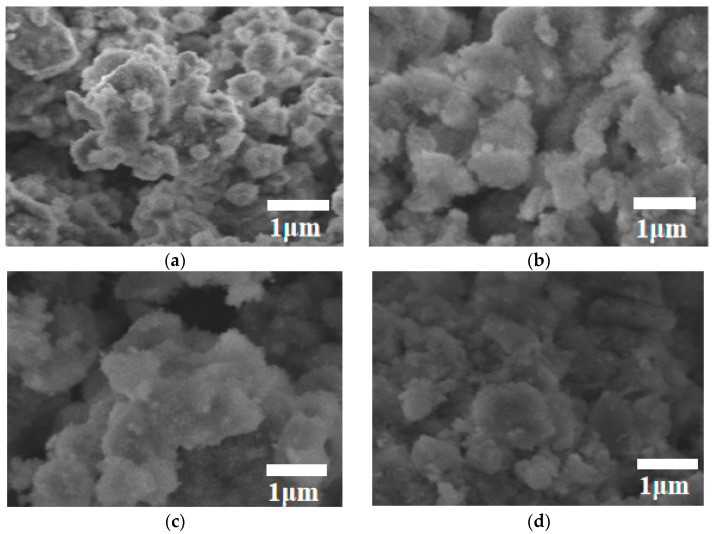
The morphology of a fly ash-based geopolymer when the foam content is 0 g (**a**), 15 g (**b**), 20 g (**c**) and 25 g (**d**), respectively.

A large number of fibrous cementitious materials accumulate into a near-spherical structure as shown in Figure 5a–d. This is because the Si-O-Al covalent bond of highly active metakaolin is firstly destroyed in the reaction process, it combines with alkaline substances to form a gel, which attaches to the surface of spherical fly ash particles. As the degree of carburization deepens and the degree of polymerization increases, the nearly spherical particles gradually become larger, and the density of the structure gradually increases. Finally, a unique three-dimensional network structure is formed, forming the framework of fly ash-based geopolymers.

## 4. Conclusions

The 1 h settlement distance, average foaming multiple and bleeding ratio of foam prepared by the bio-based foaming agent (0.50 wt% CTAB and 10% yeast solution) are 15 mm, 36.7-fold and 62.2%, respectively, which fully meet the preparation of lightweight geopolymers. With the increase in foam content, the dry density, thermal conductivity and compressive strength of the lightweight geopolymer gradually decrease, and the water absorption increases when using the above bio-based foaming agent to prepare the geopolymer, regardless of whether the raw material is silica fume or fly ash. When the content of foam is 25 g, the dry density of the fly ash-based geopolymer is reduced to 678.4 kg/m^3^ and the thermal conductivity is reduced to 0.073 W/(m·K), but its compressive strength can still be maintained at 6.4 MPa. Therefore, the bio-based foaming agent prepared by the combination of yeast and CTAB effectively improves the defects of traditional foaming agents, and provides a novel idea for the development of bio-based lightweight geopolymers. However, the durability of lightweight geopolymers, such as freeze–thaw cycle resistance, carbonation resistance, sulfate resistance, chloride resistance, alkali–silica reactions and so on, will be further studied.

## Figures and Tables

**Table 1:** The mix design of the lightweight geopolymer.

Metakaolin/g	Silica Fume/g	Fly Ash/g	Alkali Activator/g	Water-Reducing Agent/g	Water/g	Foam Content/g
252	108	---	140	2.5	220	0
252	108	---	140	2.5	220	20
252	108	---	140	2.5	220	25
252	108	---	140	2.5	220	30
128	---	282.5	111.5	2	200	0
128	---	282.5	111.5	2	200	15
128	---	282.5	111.5	2	200	20
128	---	282.5	111.5	2	200	25

## Data Availability

The original contributions presented in the study are included in the article, further inquiries can be directed to the corresponding author.
